# Deletion of *relA* abrogates the capacity of *Mycobacterium avium paratuberculosis* to establish an infection in calves

**DOI:** 10.3389/fcimb.2014.00064

**Published:** 2014-05-15

**Authors:** Kun Taek Park, Andrew J. Allen, George M. Barrington, William C. Davis

**Affiliations:** ^1^Department of Microbiology and Pathology, College of Veterinary Medicine, Washington State UniversityPullman, WA, USA; ^2^Department of Veterinary Clinical Sciences, College of Veterinary Medicine, Washington State UniversityPullman, WA, USA

**Keywords:** *Mycobacterium avium* subsp. *paratuberculosis*, *relA*, immune response, IL-12R, IL-23R, regulatory T cells

## Abstract

Previous comparative studies in goats revealed deletion of *relA* but not *pknG* abrogates the capacity of *Mycobacterium avium* subsp. *paratuberculosis* (*Map*) to establish a persistent infection. The immune response elicited by the mutant cleared infection. The objective of the present study was to extend the studies in calves and compare the proliferative response elicited by the *relA* deletion mutant (Δ*relA*) and *Map* using flow cytometry and quantitative reverse transcription real-time PCR (qRT-PCR). Six 3-day-old calves were divided into two groups. Three were vaccinated with Δ*relA* and 3 inoculated with wild type *Map*. The calves were challenged with *Map* 1 month later and necropsied 3 months post challenge. Three untreated calves were used as uninfected controls. Examination of tissues revealed the Δ*relA* mutant was immune eliminated. Bacterial load of *Map* was significantly reduced in the calves vaccinated with Δ*relA* and challenged with *Map* in comparison with calves inoculated and challenged with *Map*. A vigorous CD4 memory T cell response was detected at necropsy in PBMC from both infected groups. CD8 positive NK cells proliferated in the presence and absence of antigen stimulation in both treated groups but not in the uninfected group. IFN-γ, IL17, and IL22 gene expression were up-regulated with an associated increase in their transcription factors, Tbet and RORC, in both treated groups. TGF-β, IL-10, and FoxP3 were not up-regulated, indicating no activation of regulatory T cells. The findings show that the immune response to Δ*relA* is clearly different than the response to *Map*. Understanding the immunological basis for this difference should facilitate development of a vaccine that elicits sterile immunity.

## Introduction

As part of an international effort to develop a vaccine that prevents or limits the capacity of *Mycobacterium avium* subsp. *paratuberculosis* (*Map*) to establish a persistent infection and cause disease, we focused on use of targeted allelic exchange mutagenesis to identify genes essential for establishment of a persistent infection. We selected 3 genes to initiate the studies and optimize use of allelic exchange mutagenesis with slow growing mycobacteria: *relA* (a global regulator), *pknG* (a gene encoding a kinase that interferes with phagosome lysosome fusion), and *lsr2* (a gene regulating lipid biosynthesis and antibiotic resistance) (Park et al., 2008). Our first studies revealed the efficiency of allelic exchange transduction, with a mycobacterial phage containing an allelic exchange substrate, could be enhanced by allowing aggregates of *Map* to sediment out of the culture to obtain a cell preparation comprised of single cells and by increasing the selective pressure with hygromycin (Park et al., 2008). Subsequent studies revealed deletion of these genes impaired survival in macrophages *ex vivo* in comparison with survival of *Map*, suggesting deletion of any of these genes might impair survival *in vivo*. Based on these findings, the mutants were submitted to the JDIP-APHIS Vaccine Testing Program for further evaluation along with other potential vaccine candidates. Studies were also conducted in parallel to test the capacity of the mutants to establish an infection. Preliminary studies in calves with the *relA* and *pknG* deletion mutants (Δ*relA* and Δ*pknG*) revealed immunization with Δ*relA* elicited an immune response that cleared infection, as assessed by screening tissues for the presence of Δ*relA*, whereas, immunization with *pknG* elicited an immune response that only impaired establishment of an infection with Δ*pknG* (Park et al., 2011). A subsequent challenge study in kid goats with all 3 mutants revealed Δ*relA* elicited an immune response that cleared the mutant and impaired establishment of an infection with *Map*. Deletion of *pknG* did not prevent establishment of an infection with Δ*pknG* or impair establishment of infection with *Map* (Park et al., 2011). Deletion of *lsr2* resulted in attenuation of *in vivo* survival, but immunization with the mutant did not elicit an immune response that limited infection with *Map* (unpublished observation). The present study was conducted to verify and extend observations made with Δ*relA* using a calf challenge model. The current study includes analyses of cytokine gene expression profiles and the proliferative response of NK cells, γδ and αβ T cells, and Foxp3 regulatory T cells following stimulation with live *Map* using quantitative reverse transcription real-time PCR (qRT-PCR) and flow cytometric (FC) analysis which were not done in the previous goat challenge study.

## Materials and methods

### Animals

Six bull calves were obtained from the Johne's disease-free Washington State University dairy herd and maintained according to the protocols and procedures approved by the Washington State University Institutional Animal Care and Use Committee. The calves were taken to a Biosafety Level 2 isolation unit within the first 24 h of life and separated into groups of three. They were fed 4 L of maternally derived colostrum within 6 h of birth and subsequently fed milk replacer, whey pellets, calf starter grain, and then free choice alfalfa hay during the study. Three additional calves (untreated control group) of the same age as the experimental calves, maintained at the dairy, were used as uninfected controls.

### Preparation of bacteria, inoculation, and challenge

Cultures of *Map* and Δ*relA* were prepared as previously described (Park et al., 2011). Three calves were inoculated per os with 10^9^ *Map* K10 strain (K10 group) and three with 10^9^ Δ*relA* (RelA group) within 3 days of birth. At 1 month post inoculation they were challenged with 10^9^ *Map* K10 per os and then necropsied at 3 months post challenge. All inoculation and challenge bacteria were prepared in 1 L of milk.

### Peripheral blood mononuclear cell isolation and stimulation

Blood was collected at the time of necropsy, and peripheral blood mononuclear cells (PBMC) were isolated and cultured in RPMI-1640 medium with and without live *Map* as previously described (Park et al., 2011). PBMC were processed for transcriptional analysis of cytokine genes at day 3 and for FC analysis at day 6 of culture as described below.

### Flow cytometry

Monoclonal antibodies (mAb) specific for NK (CD335, IgG1), TCR1 δ chain (GB21A, IgG2b), CD2 (MUC2A, IgG2a), CD4 (ILA11A, IgG2a), CD8 (7C2B, IgG2a), CD25 (LCTB2A, IgG3 and CACT116A, IgG1), and CD45R0 (ILA116A, IgG3) were used for FC analysis to study the response of PBMC *ex vivo* (Allen et al., 2009, 2011). A mAb specific for transcription factor FoxP3 (FOX5A, IgG1) was used to study the regulatory T cell (Tr) response (Seo et al., 2009). The intracellular labeling of FoxP3 was conducted using the Foxp3/Transcription Factor Staining Buffer Set following the manufacture's recommendation (eBioscience, CA).

### Gene expression

RNA extraction from PBMC, cDNA generation, and qRT-PCR were conducted as previously described (Park et al., 2011). The sequence information of all primers used in the current study were as previously described (Park et al., 2011). The following genes were examined for expression: IFN-γ, TGF-β, FoxP3, IL-10, IL-12p35, IL-17, IL-22, IL-23p19, and granulysin (a mycobactericidal peptide) (Dieli et al., 2001; Gansert et al., 2003).

### Detection and quantification of *Map* in tissue at necropsy

Triplicate samples of nine tissue sites were processed for *Map* culture and duplicates for quantitative real-time PCR (qPCR) detection of IS900 sequence to detect and quantify *Map* in tissues. Tissues were cultured with and without hygromycin to distinguish colonies of Δ*relA* from *Map*. An average colony forming unit (CFU) from each tissue site was obtained as previously described (Park et al., 2011). *Map* genomic DNA (gDNA) extraction from tissues was conducted as previously described (Park et al., 2014). The copy number of *Map* gDNA in the sample was quantified by IS900 qPCR (Irenge et al., 2009). *Map* gDNA copy number per 100 mg tissue of each tissue was transformed to a log scale, and compared between K10 and RelA groups for the same tissue sites. Note that the value of qPCR negative tissue (0 gDNA copy) was transformed to 0 of log value for a graph and comparison.

### Statistics

MedCalc statistical software ver. 11. 2. 1 (Belgium) was used for all statistical analyses. The frequencies of *Map* tissue culture positive sites between K10 and RelA groups were compared using Fisher's exact test. The transformed log value of *Map* gDNA copy number was used to compare the bacterial loads in each tissue site between K10 and RelA groups using Kruskal–Wallis test or One-Way ANOVA. The results of FC analysis for CD4 and CD8 T cell activation and qRT-PCR for relative gene transcriptions were analyzed using Kruskal–Wallis test or One-Way ANOVA. In all tests, a *p*-value of less than 0.05 was considered to be significant.

## Results

**Figure 1:**
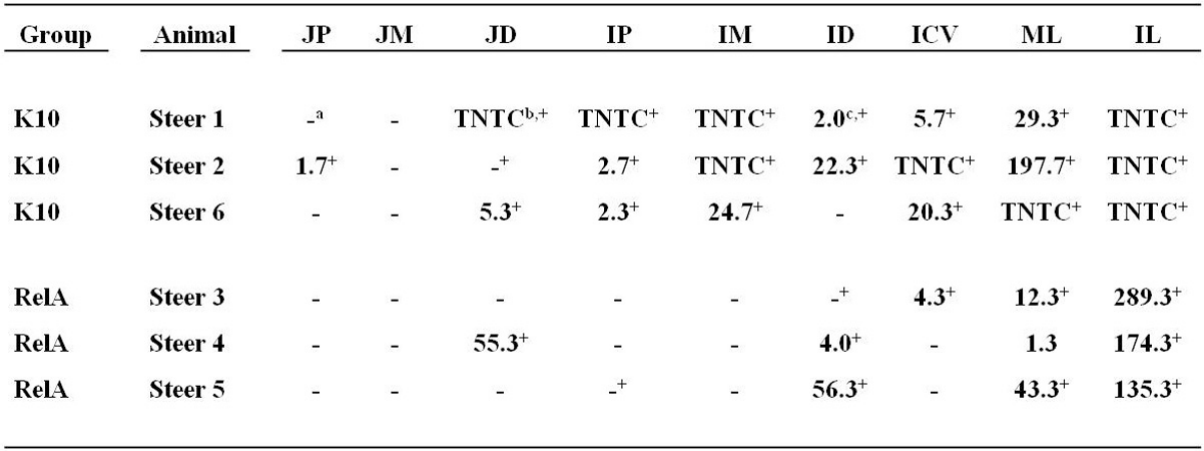
***Map* culture results from nine tissue sites processed at necropsy**. The data are expressed as the average CFU of triplicate agar cultures containing no hygromycin. No colonies were detected in the agar cultures containing hygromycin, which indicates Δ*relA* was cleared in the vaccinated animal. JP, JM, JD: jejunum proximal, middle, distal; IP, IM, ID: ileum proximal, middle, distal; ICV: ileocecal valve; ML, IL: mesenteric and ileocecal lymph nodes. a, Negative; b, too numerous to count; c, Values are expressed as the average CFU obtained from three agar cultures; +, positive by IS900 qPCR.

**Figure 2:**
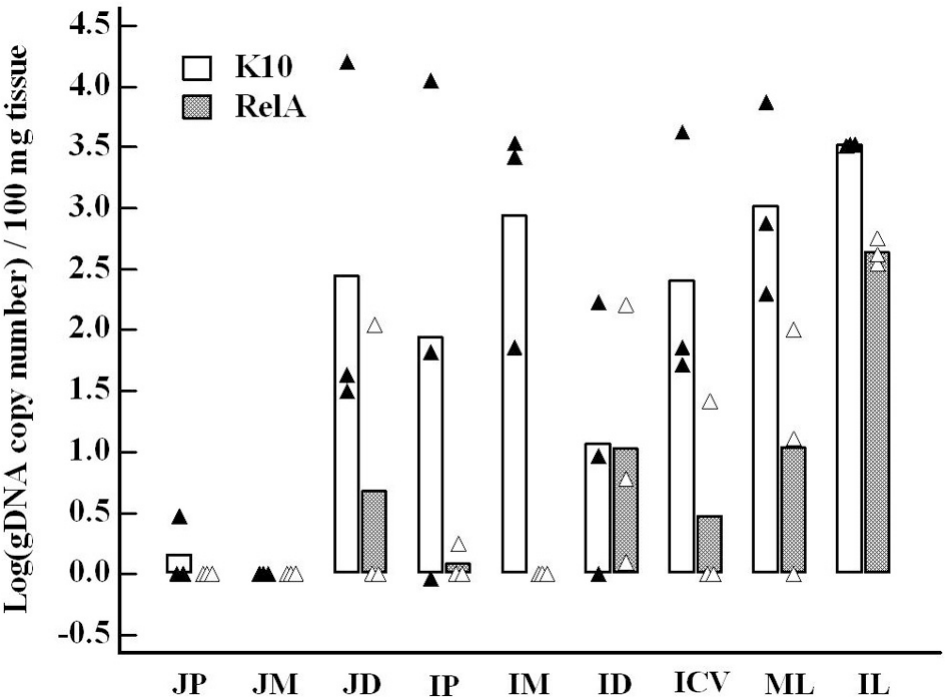
**Comparison of bacterial loads in each tissue site between K10 and RelA groups**. *Map* gDNA copy number per 100 mg tissue of each sample measured by IS900 qPCR was transformed to a log scale. The results were compared for the same tissue site between the two groups. Data are presented as the average of three animals of each group (bar graph) with all individual data (K10 group, closed triangle, and RelA group, open triangle, respectively). JP, JM, JD: jejunum proximal, middle, distal; IP, IM, ID: ileum proximal, middle, distal; ICV: ileocecal valve; ML, IL: mesenteric and ileocecal lymph nodes. The bacterial loads in IM and IL were statistically significant between the two groups (*p* < 0.05).

No colonies of Δ*relA* were detected in any of the tissues from calves inoculated with Δ*relA* (Figure 1). The frequency of *Map* culture positive tissues was reduced in the RelA group (10 positive tissues out of 27 screened tissues, 37.0 %) in comparison with that in K10 group (20/27 positive tissues, 74.1 %) (*p* < 0.05, Figure 1). To compare the bacterial loads in the tissues, *Map* gDNA copy number per 100 mg tissue of each tissue sample was calculated using IS900 qPCR since the exact CFU information of many tissue sites of K10 group were not available due to the excess of countable ranges (too numerous to count, TNTC). We previously demonstrated an excellent correlation between the quantification of *Map* gDNA in tissues by qPCR and CFU obtained by *Map* culture from the samples (Park et al., 2014). The average gDNA copy numbers of the RelA group were higher than K10 group for all tissue sites processed, except the tissue samples of middle jejunum (all negative in both groups). For 5 tissue sites (distal jejunum, proximal ileum, middle ileum, ileocecal valve, and mesenteric lymph node) the differences of bacterial loads between the two groups were more than 1.5 logs per 100 mg tissue (Figure 2).

**Figure 3:**
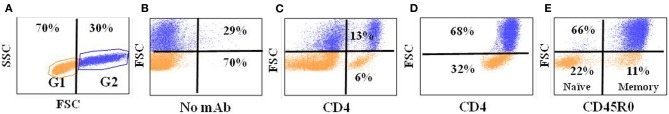
**Gating strategy used to analyze the proliferative response of peripheral blood mononuclear cells (PBMC) *ex vivo* following stimulation with live *Map***. Representative CD4 profiles obtained with PBMC from a calf inoculated/vaccinated with Δ*relA* and challenged with wild type *Map*. **(A)** Selective gates were placed on resting/unstimulated cells (G1) and stimulated proliferating cells (G2) and color coded to track activated cells. **(B)** Cells with no mAb. **(C)** Cells labeled with CD4 with no gate to show relative percent of resting and activated CD4 cells. **(D)** CD4 cells with a gate to select CD4 cells for analysis. **(E)** CD4 cells showing expression of CD45R0 only on memory cells. The activated cells (blue) also expressed CD25.

**Figure 4:**
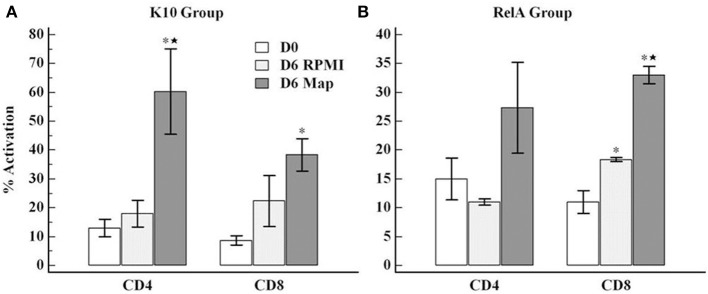
**Flow cytometric analysis of the proliferative response of PBMC cultured *ex vivo* in absence/presence of live *Map***. The figure shows the frequency of activated CD4 (CD4/CD25/CD45R0 positive) or CD8 (CD8/CD25/CD45R0 positive) cells obtained with PBMC from the K10 group **(A)** and the *relA* group **(B)**. The data represent the mean percent activation of CD4 and CD8 T cells with error bar (SE). D0, PBMC at day 0; D6 rpmI and D6 *Map*, PBMC cultured in medium alone and with live *Map* for 6 days, respectively; ^*^ and ⋆, significant difference compared to D0 and D6 rpmI, respectively (*p* < 0.05).

**Figure 5:**
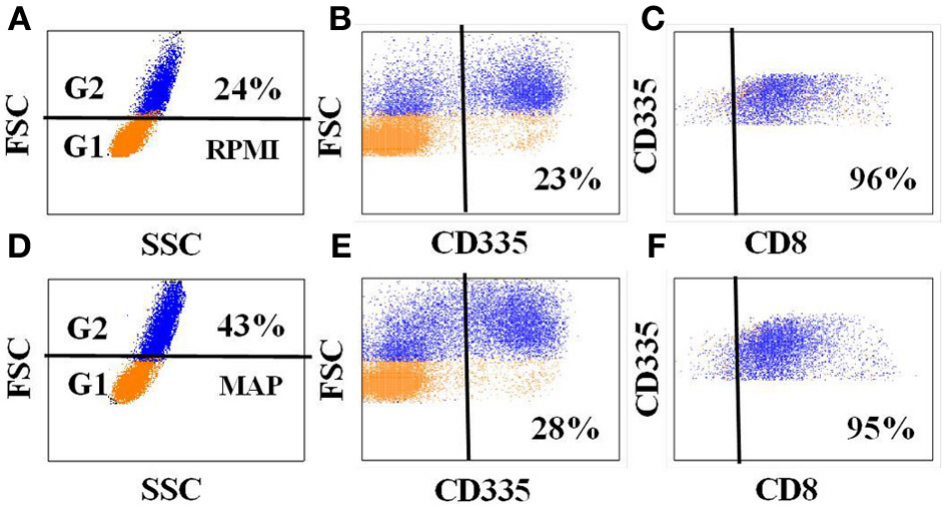
**Expression of CD8 on NK cells in PBMC from *Map* infected animals**. Data show the expression of CD8 on NK cells in PBMC cultured in medium alone **(A–C)** and in the presence of *Map* **(D–F)**. An additional gate was placed on NK cells to show co-expression of CD8 **(C,F)**.

A representative gating strategy used for FC analysis of fresh and cultured PBMC is shown in Figure 3. CD4 T cells proliferated in response to stimulation with live *Map* and expressed CD25 and CD45R0 (Figures 3, 4). By using a negative electronic gate to exclude CD8 positive NK cells it was possible to show CD8 positive αβ T cells also proliferated in response to *Map* stimulation (Figure 4). FC analysis of NK cells revealed NK cells were activated and proliferated in culture medium alone and in the presence of *Map*. The majority of activated NK cells expressed CD2 and CD8 (Figure 5, data only shown for CD8). WC1 positive and WC1 negative γδ T cells were not activated in this study (data not shown).

**Figure 6:**
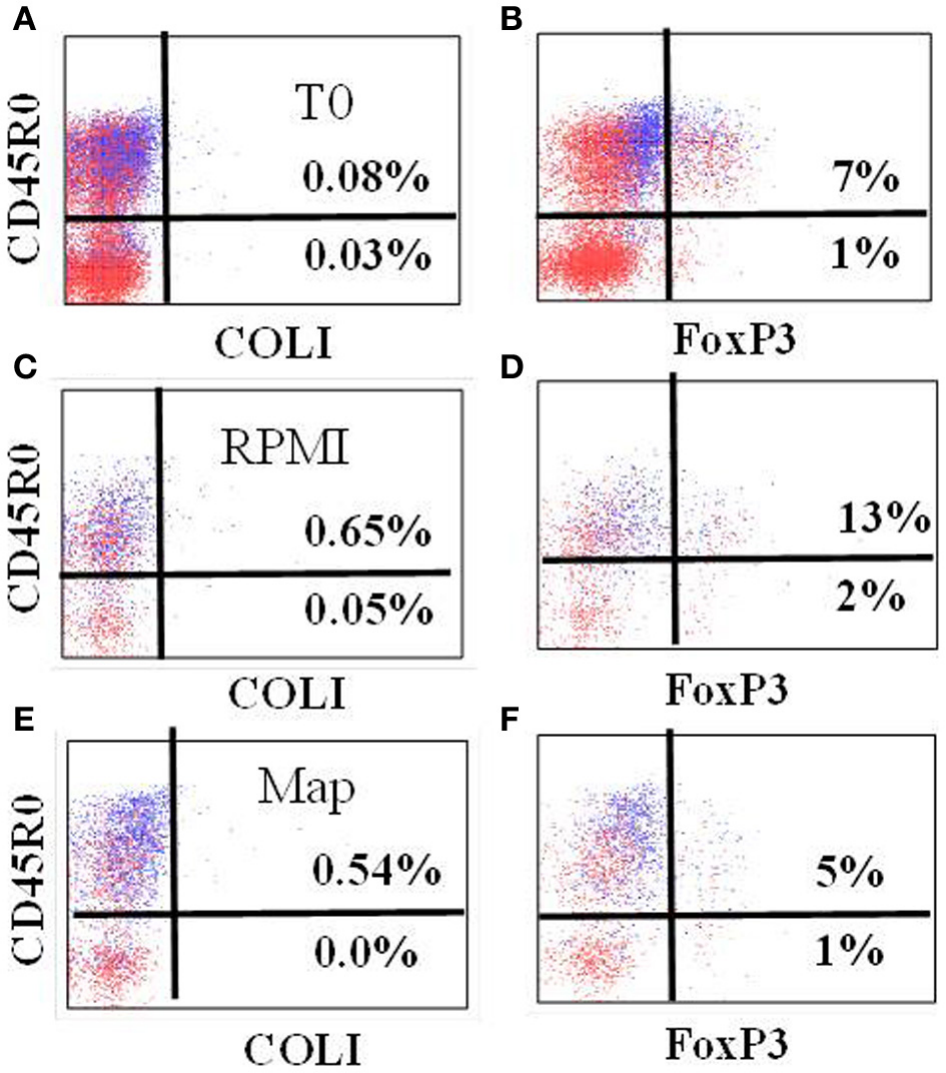
**Representative profiles showing expression of FoxP3 in gated CD4 T cells at T0 (A,B), and cultured in RPMI alone (C,D) and stimulated with live *Map* (E,F) from a calf of RelA group**. As noted, use of anti-CD45R0 rather than anti-CD25 provides better resolution of cells expressing FoxP3. COLI = isotype control.

FC analysis of FoxP 3 (Figure 6) showed the proportion of CD4 T cells expressing FoxP3 was low at initiation of culture in calves in the K10 group (2.4% ± 0.5 SE) and calves in the RelA group (4.7% ± 1.2 SE). The proportion of CD4 T cells expressing FoxP3 increased in cells cultured in medium alone (K10 group: 4.0% ± 0.3, and RelA group: 11.0% ± 2.6, respectively) while there was minimal change in cells cultured with live *Map* (K10 group: 2.0% ± 1.1, and *relA* group: 6.0 ± 0.9%, respectively). The overall proportion of Foxp3 expressing CD4 T cells was slightly higher in the RelA group than in the K10 group.

**Figure 7:**
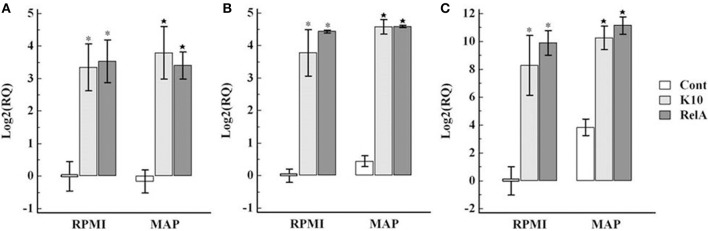
**Relative transcription of genes involved in Th1 type immune response in PBMC**. PBMC were isolated from animals in each group and cultured with and without live *Map* for 3 days. The level of mRNA transcription of PBMC was measured by qRT-PCR. The relative quantification of each cytokine was calculated using the value of PBMC isolated from control animals (uninfected animals) and cultured in RPMI medium alone as the calibrator. The panels show the relative quantification of IL-12p35 **(A)**, Tbet **(B)**, IFN-γ **(C)**. RQ, relative quantification; RPMI and *MAP*, PBMC cultured in RPMI medium alone and with live *Map*, respectively; ^*^ and ⋆, significant difference compared to RPMI and MAP of the control group, respectively.

**Figure 8:**
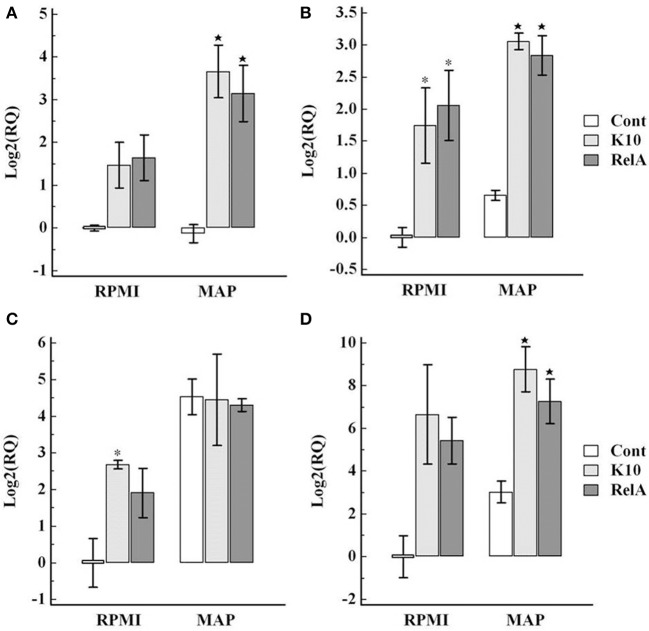
**Relative transcription of genes involved in Th17 type immune response in PBMC**. The relative quantification of mRNA transcription was determined as described in the legend of Figure 7. The panels show the relative quantification of IL-23 **(A)**, RORC **(B)**, IL-17 **(C)**, IL22 **(D)**. RQ, relative quantification; RPMI and *MAP*, PBMC cultured in RPMI medium alone and with live *Map*, respectively; ^*^ and ⋆, significant difference compared to RPMI and MAP of the control group, respectively.

Analysis of expression of cytokines associated with the Th1 (IL-12p35, IFN-γ, and transcription factor Tbet) and Th17 (IL-23, IL-17, and IL-22, and transcription factor RORC) axes in cultured PBMC showed there was an increase in expression in both K10 and RelA groups in cells cultured in medium alone and in the presence of *Map* in comparison with the uninfected control group (Figures 7, 8).

**Figure 9:**
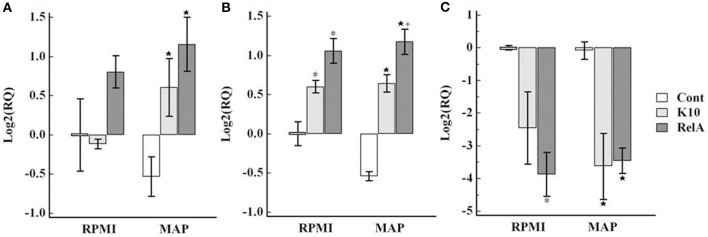
**Relative transcription of genes involved in regulating the immune response in PBMC**. The relative quantification of mRNA transcription was determined as described in the legend of Figure 7. The panels show the relative quantification of FoxP3 **(A)**, TGF-β **(B)**, IL-10 **(C)**. RQ, relative quantification; RPMI and *MAP*, PBMC cultured in RPMI medium alone and with live *Map*, respectively; ^*^ and ⋆, significant difference compared to RPMI and MAP of the control group, respectively; ^+^, significant difference compared to MAP of K10 group.

The regulatory cytokine gene responses in PBMC varied. In comparison with the uninfected control group, the transcription factor (FoxP3) and TGF-β were slightly up-regulated, but the expression of IL-10 was highly down-regulated in the infected groups (Figure 9).

**Figure 10:**
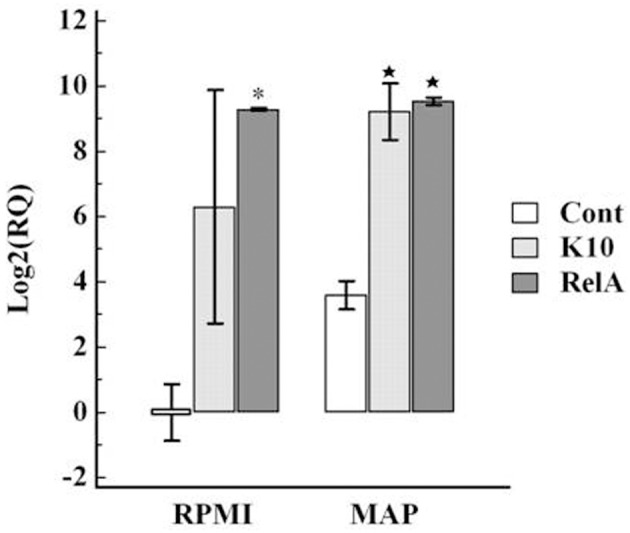
**Relative transcription of the granulysin gene in PBMC**. The relative quantification of mRNA transcription was determined as described in the legend of Figure 7. RQ, relative quantification; RPMI and *MAP*, PBMC cultured in RPMI medium alone and with live *Map*, respectively; ^*^ and ⋆, significant difference compared to RPMI and *MAP* of the control group, respectively.

The expression of granulysin, a mycobactericidal peptide, was slightly up-regulated against *Map* stimulation in the control group. However, the up-regulation was more significant in the challenged groups (Figure 10).

## Discussion

Methods for analysis of the effect of gene disruption on the immune response to *Map* remain a problem. The current strategy has been to examine the effect of mutation on survival in macrophages, then screen selected mutants for survival in mice and their ability to elicit an immune response that prevents or limits establishment of infection, and finally test promising mutants for efficacy in one of the natural hosts. Recent studies have emphasized the limitations of this approach and the potential of missing the best candidates for analysis (Scandurra et al., 2010; Park et al., 2011). A reduction in the capacity to survive in macrophages does not necessarily predict survival *in vivo* or the immune response to the mutant. Testing in an animal model, like the mouse, is more direct but it does not necessarily predict the immune response in the natural host (Scandurra et al., 2010). Clearly, the more reliable method would be to examine the immune response in the natural host. Even here, the ability to study the effect of gene deletion on the immune response is limited. Necropsy and examination of tissues is needed to determine if deletion of a gene abrogates the capacity of *Map* to subvert the immune response and evade immune elimination. Although arduous, this approach does provide a means for identifying candidate mutants for further evaluation.

Results from the first round of screening candidate mutants in macrophages for inclusion in the JDIP-APHIS Vaccine Testing Program, suggested none of the mutants we developed, including the Δ*relA* mutant, were candidates for inclusion and further evaluation. This emphasizes the difficulty of using an indirect method of screening mutants for their potential as a vaccine. Of the three mutants we submitted to the program, Δ*relA* is the only mutant shown to elicit an immune response that clears infection and limits colonization of *Map* under experimental challenge conditions. This response is clearly different than the immune response elicited by wild type *Map*. Exposure to Δ*relA*, per os at birth or through direct inoculation into the ileum through a cannula, elicits a response that clears infection of the mutant and limits the capacity of *Map* to colonize and establish an infection (Park et al., 2011). In contrast, exposure to *Map* by either route elicits a response that does not affect colonization.

How deletion of *relA* disrupts the pathway(s) involved in modulating the immune response remains to be elucidated. Comparison of the immune response to Δ*relA* and *Map* has thus far, not revealed any clear differences between the response to Δ*relA* and *Map*. The results have also revealed *ex vivo* analysis of the immune response in calves vaccinated with Δ*relA* is comparable to the response developed in calves inoculated with *Map*. The response is characterized by a CD4 and CD8 proliferative response to PPD, soluble antigens, and live *Map* (Koo et al., 2004). With the limited number of animals examined thus far, it is difficult to determine if there is a significant difference in the proliferative response to *Map* in calves inoculated with *Map* or Δ*relA*. In our previous study, live *Map* stimulation appeared to induce a more vigorous CD8 cell response than the CD4 cell response in PBMC from the wild type *Map* and Δ*relA* inoculated groups (Park et al., 2011). However, as shown here, the NK CD8 positive cell subset from animals exposed to *Map* proliferate nonspecifically. This population couldn't be distinguished from αβ CD8 cells in the previous study. This population most likely accounted for the increase noted in our earlier study. γδ T cells did not proliferate in this study.

Although the frequency was slightly higher in RelA group than in K10 group, the pattern of FoxP3 expression in CD4 T cells was similar in both challenged groups. The frequency was low in the circulating PBMC (at Time 0). The frequency was similar after stimulation with *Map*, but increased in cultures without *Map*. The interpretation should be done with the results of the CD4 T cell proliferation responses (Figure 4). CD4 T cells proliferated vigorously in both groups when cultured in the presence of *Map*, but not in medium alone. Foxp3 was expressed only in a subset of the memory T cell population (CD25+ and CD45R0+). Therefore, the apparent increase in the frequency of Foxp3 expressing cells in medium alone could be attributed to the change in proportion of naïve and activated proliferating CD4 T cells, not to proliferation of the Tr cells. Likewise, the frequency of Foxp3 cells in cultures with *Map* may have appeared unchanged owing to the fact that proliferation of Tr cells and effector T cells was proportional following stimulation. This supposition is supported by the transcription analysis results which showed the expression of Foxp3 was slightly higher in cell cultured with *Map* compared to cells cultured in medium alone in both groups (Figure 9). The other regulatory cytokines (TGF-β and IL-10) were not up-regulated in both animal groups compared to the control group. These finding indicate there was no Tr mediated inhibitor effect detected in the present study. It would be expected that there would be very little activity during the initial response to *Map* when infection is under immune control. Later stages of infection need to be examined to determine if Tr cells play a role in modulating the immune response to *Map*.

Interpretation of other cytokine gene expression profiles is difficult because of the non-specific proliferation of the CD8 positive NK cell subset and expression of cytokine genes in medium alone and in the presence of *Map*. The transcription of cytokines associated with Th1 response, IL-12p35 and IFN-γ, and the Th17 response, IL23, IL17, and IL-22, were up-regulated in both infected groups when cultured in medium alone in comparison with values obtained with cells from the control group. The expression levels were slightly elevated when stimulated with live *Map*. Inherent increase of IFN-γ and granulysin gene expression in unstimulated cells from *Map* infected cows and further increase in response to live *Map* stimulation has been observed in previous studies (Coussens et al., 2004; Park et al., 2011). The current study extends the observation to other cytokines associated with Th1 and Th17 type immune responses. Further studies are needed to explain these observations. These cytokines can also be produced by other types of immune cells, such as NK cells. NK cells proliferated in the presence and absence of *Map* stimulation, potentially explaining increased expression in medium alone. The increase of IL-17 mRNA transcription was also observed in PBMC from uninfected animals when stimulated with live *Map*. However, this stimulation did not induce an increase in IL-23 or IL-22.

In summary, the present study has provided further data showing deletion of *relA* abrogates the ability of *Map* to establish a persistent infection. Exposure/vaccination with Δ*relA* elicited an immune response that cleared the infection with Δ*relA*. This is in contrast to the other two mutants we selected for evaluation. Studies with *Mycobacterium bovis* (*Mbv*), *pknG* in *Mbv* BCG and in *M. smegmatis* (with the inserted gene) showed the enzyme was essential for survival in macrophages (Walburger et al., 2004). This strongly suggested that, if survival in macrophages was an important predictor of survival *in vivo*, deletion of *pknG* in *Map* should affect the survival of *Map in vivo*. The Δ*pknG* mutant of *Map* showed a similar result to that of the *pknG* deletion mutant in *Mbv* BCG in bovine monocyte-derived macrophages. The survival of Δ*pknG* was significantly reduced compared to wild type *Map* and Δ*relA*. However, the *in vivo* survival of Δ*pknG* was not attenuated in calves and kid goats (Park et al., 2011). Lsr2 was initially known to be associated with lipid biosynthesis in mycobaterial cell wall (Chen et al., 2006). More recent studies revealed that it is a global regulator which regulates expression of multiple genes, including genes associated with cell wall biosynthesis, antibiotic resistance, and mycobacterial pathogenesis (Chen et al., 2008; Gordon et al., 2010; Liu and Gordon, 2012). In comparison to Δ*pknG*, the *lsr2* deletion mutant of *Map* showed less attenuation in macrophages, but significant attenuation in kid goats. However, the Δ*lsr2* did not elicit an immune response that affected establishment of infection with *Map* under experimental challenge conditions (unpublished data). The results from *ex vivo* and *in vivo* studies with these mutants provide further evidence that survival in macrophages is not a good predictor of survival *in viv*o or the capacity of the mutants to elicit a response that clears infection. Studies in a mouse model showed deletion of *relA* in *Mtb* impaired the capacity of *Mtb* to survive *in vivo*. It was hypothesized that deletion of *relA* is critical for successful establishment of a persistent infection and that deletion of *relA* alters expression of antigenic and enzymatic factors essential for establishment of a latent infection (Dahl et al., 2003). The data obtained in our studies support this hypothesis. Further studies are needed now to identify the enzymatic pathways and their expressed products that are altered by deletion of *relA*.

## Author contributions

All authors were involved in the design of the experiments. William C. Davis and Kun Taek Park were responsible for overseeing the conduct of the studies and analysis of the data.

### Conflict of interest statement

The authors declare that the research was conducted in the absence of any commercial or financial relationships that could be construed as a potential conflict of interest.
